# Development and Characterization of Field Structured Magnetic Composites

**DOI:** 10.3390/polym13172843

**Published:** 2021-08-24

**Authors:** Balakrishnan Nagarajan, Yingnan Wang, Maryam Taheri, Simon Trudel, Steven Bryant, Ahmed Jawad Qureshi, Pierre Mertiny

**Affiliations:** 1 Department of Mechanical Engineering, University of Alberta, Edmonton, AB T6G 1H9, Canada; bnagaraj@ualberta.ca (B.N.); yingnan1@ualberta.ca (Y.W.); ajqureshi@ualberta.ca (A.J.Q.); 2 Department of Chemistry, University of Calgary, Calgary, AB T2N 1N4, Canada; maryam.taheri2@ucalgary.ca (M.T.); trudels@ucalgary.ca (S.T.); 3 Department of Petroleum and Chemical Engineering, University of Calgary, Calgary, AB T2N 1N4, Canada; steven.bryant@ucalgary.ca

**Keywords:** magnetic polymer composites, anisotropic properties, dual-cure resin, polymer casting, additive manufacturing

## Abstract

Polymer composites containing ferromagnetic fillers are promising for applications relating to electrical and electronic devices. In this research, the authors modified an ultraviolet light (UV) curable prepolymer to additionally cure upon heating and validated a permanent magnet-based particle alignment system toward fabricating anisotropic magnetic composites. The developed dual-cure acrylate-based resin, reinforced with ferromagnetic fillers, was first tested for its ability to polymerize through UV and heat. Then, the magnetic alignment setup was used to orient magnetic particles in the dual-cure acrylate-based resin and a heat curable epoxy resin system in a polymer casting approach. The alignment setup was subsequently integrated with a material jetting 3D printer, and the dual-cure resin was dispensed and cured in-situ using UV, followed by thermal post-curing. The resulting magnetic composites were tested for their filler loading, microstructural morphology, alignment of the easy axis of magnetization, and degree of monomer conversion. Magnetic characterization was conducted using a vibrating sample magnetometer along the in-plane and out-of-plane directions to study anisotropic properties. This research establishes a methodology to combine magnetic field induced particle alignment along with a dual-cure resin to create anisotropic magnetic composites through polymer casting and additive manufacturing.

## 1. Introduction

Magnetic composites are fundamental elements in many electrical, electronic and electromagnetic devices. Mixing magnetic powders with a polymeric binder that bonds and insulates the powder grains enables manufacturing magnetic composites for a variety of applications. Advantages of magnetic composites include the ability to be produced with complicated shapes, high dimensional accuracy, and good mechanical, magnetic, and physical properties. Magnetic composites are divided into hard magnetic and soft magnetic composites [1]. Hard magnetic composites, also known as polymer bonded permanent magnets, find significant applications in motors, sensors and actuators in automation equipment and medical devices. Common methods utilized to manufacture bonded magnets include compression molding, injection molding, calendaring and extrusion [2,3,4]. The properties of magnetic composites depend on the powder particle size and shape, type of polymeric binder, and the manufacturing process utilized [5]. Field structured magnetic composites fabricated by applying an external magnetic field during the polymerization process exhibited high remanence due to local field effects. It was observed that a uniaxial field produced chain-like particles, and a biaxial field produced sheet-like particle structures in the fabricated composites [6]. Composites with magnetostrictive features were developed using an injection molding method where carbonyl iron particles were field structured in a thermoplastic elastomer matrix using an electromagnet. It was observed that samples with aligned iron particles exhibited a higher modulus compared to randomly dispersed magnetic polymer composites [7].

Functional magnetic composites, magnetic shape memory alloys, magnetic micro electromechanical systems (MEMS) and magnetic elastomers use a variety of magnetic materials [8]. Magnetic forces offer an attractive option for actuation in MEMS devices due to contact-free actuation capabilities. Microscale magnetic actuation capabilities have led to the implementation in a variety of microfluids and MEMS devices. In the field of micro-robotics, magnetic forces are used to provide wireless control and power to perform complex three-dimensional motions. Integration of different magnetic fillers with the polymer resin remains a significant challenge in the fabrication process [9]. Fabrication of micro magnets using traditional ultraviolet light (UV) lithography and micro-molding techniques have already been reported in the technical literature. However, technical challenges like adjusting suspension viscosity for spin-coating processes, particle settling, and precise control of particle alignment still exist in the fabrication of magnetically loaded polymer composites for microscale applications [10]. A micro pump with diffuser elements and an integrated composite magnet was developed using neodymium iron boron (NdFeB) magnet powder dispersed in polydimethylsiloxane (PDMS) resin [11]. High-performance NdFeB micro magnets using magnetron sputtering and high power plasma etching techniques have been reported in the technical literature [12]. Using low-modulus membrane materials, elastic hard magnetic films with the ability to produce bi-directional deflections in an external magnetic field were created using microfabrication approaches [13]. Mechanically compliant, magnetically responsive micro structures using a ferromagnetic photoresist containing nickel nanospheres dispersed in photosensitive epoxy resin (SU8) were fabricated using UV lithography-based approaches [14]. Screen printing was used as a technique to fabricate strontium ferrite thick films with an easy axis orientation perpendicular to the film surface [15]. Hard magnetic films were fabricated by embedding anisotropic NdFeB particles in a polymethylmethacrylate polymer matrix for MEMS applications. Fabricated thick films exhibited out of plane macroscopic magnetic anisotropy [16]. The immense capabilities of magnetic field responsive materials in terms of magnetic actuation, deformation capabilities like stretching, bending, and rotation upon exposure to magnetic fields, abilities for controllable drug release, and shape memory behavior have made polymer based magnetic materials a topic of intensive research [17]. Even though many robust manufacturing approaches exist, new innovations in materials and manufacturing processes can significantly enhance the performance of many functional devices. One such fast emerging technology is additive manufacturing, which will be detailed in the following.

Additive manufacturing (AM) is gaining momentum towards developing bonded magnets and magnetic composites with a wide range of magnetic particles and polymeric matrix materials. The fabrication of bonded magnets by adopting material jetting AM processes with epoxy resin based magnetic pastes was demonstrated in the technical literature [18,19,20]. Rheological additives and multimodal magnetic mixtures were utilized to develop the feedstock materials for material jetting AM processes. A multi extruder 3D printer was utilized by Yan et al. to fabricate magnetic components for power electronics applications [21]. Magnetic material formulations developed using UV curable resins were printed using nScrypt tabletop micro dispensing equipment that enabled the precise control of printed layer thickness of the deposited material [22,23]. Manufacturing of permanent magnets using extrusion-based AM of thermoplastic based feedstock materials has been detailed in the technical literature [24,25,26,27]. The ability of thermoplastic materials to be remelted under the influence of heat enabled conducting in situ magnetic alignment studies of NdFeB alloy powder reinforced ethylene vinyl acetate co-polymer-based permanent magnets. It was observed that the alignment temperature, magnetic field strength, and properties of the polymer matrix influenced the degree of particle alignment [28]. Anisotropic NdFeB/samarium ferrite nitride based bonded magnets were developed adopting a post printing alignment approach using a vibrating sample magnetometer, leading to an enhancement in magnetic properties in magnetic field aligned samples [29]. Similar magnetic field aligned bonded magnets were developed using anisotropic NdFeB powder reinforcing a polyamide matrix [30,31]. In-situ magnetic alignment using an electromagnet integrated with fused deposition modelling equipment was tested on anisotropic NdFeB/samarium ferrite nitride powders reinforcing a Nylon 12 matrix. It was found that the degree of alignment was a function of applied magnetic field and printing temperature [32]. It was additionally noted that alignment studies were primarily conducted in thermoplastic polymer-bonded permanent magnets.

In this research the authors modified a UV curable acrylate-based prepolymer for heat-initiated polymerization. An in-house developed permanent magnet-based particle alignment system aimed to orient magnetic particles within the polymer matrix was tested with the dual-cure acrylate-based resin and a commercially available epoxy prepolymer. The epoxy resin was included in the present study as a baseline due to its well-established material characteristics. Experiments were initially conducted through polymer casting where 3D printed molds were utilized to hold the liquid prepolymer. The alignment magnetic jig was further integrated with material jetting based 3D printing equipment, and the dual-cure acrylate-based prepolymer was used to 3D print magnetic composites. Scanning electron microscopy was employed to observe the distribution of magnetic particles within the polymer matrix. X-ray diffraction was utilized to evaluate the orientation of the easy axis of magnetization in the magnetic composites. Samples were tested for in-plane and out-of-plane magnetic properties using a vibrating sample magnetometer. Findings from this study are expected to advance the fabrication of anisotropic magnetic thermoset polymer composites for applications in sensors and other electrical and electronic devices.

## 2. Materials

For this research, anisotropic Nd_2_Fe_14_B (herein abbreviated as NdFeB) powder of type MQA-38-14 was purchased from Magnequench Inc. (Pendleton, IN, USA). The UV curable urethane acrylate prepolymer PR48 was obtained from Colorado Photopolymer Solutions (Boulder, CO, USA). To induce heat-initiated free radical polymerization, the thermal initiator 2,2′-Azobis(2-methylpropionitrile), generally referred to as AIBN, was purchased from Sigma Aldrich (St. Louis, MO, USA). For the epoxy resin, EPON 826 with the aromatic amine curing agent Epicure W were procured from Hexion Inc. (Columbus, OH, USA). The permanent magnets used to construct the magnetic alignment array, N52 grade NdFeB magnets, were purchased from K&J Magnetics, Inc. (Pipersville, PA, USA).

## 3. Experimental Methods

### 3.1. Dual-Cure Resin Formulation Preparation and Testing

Exposing the magnetic particle reinforced prepolymer to UV, due to opacity, resulted in solidification of only the topmost surface and leaving the underlying material in an uncured state. This observation motivated the development of a formulation with the capability to cure under the influence of both UV and heat. A measured quantity of the thermal initiator was added to the UV curable prepolymer and mechanically stirred using an impeller. The resultant mixture was allowed to rest for a day prior to adding the magnetic filler to allow complete dissolution of the thermal initiator. The prepared prepolymer was transferred to small aluminum cuvettes and subsequently exposed to UV and further cured at approximately between 50 °C to 70 °C in a gravity convection oven.

### 3.2. Fabrication of Magnetic Field Structured Composites Using Polymer Casting and AM

In this study, the ability of a permanent magnet-based particle alignment fixture to orient magnetic particles in prepolymer formulations was tested. Two different material formulations namely, NdFeB powder with approximately 80 wt% dispersed in the dual-cure acrylate-based resin and the epoxy resin were utilized in this work. Small molds of dimensions 15 mm by 15 mm by 10 mm, 3D printed using a stereolithography printer, were utilized to hold the prepolymer formulation during the curing and alignment process. The oven temperature for curing the epoxy resin was between 60 °C to 80 °C.

To 3D print the dual-cure resin and develop anisotropic magnetic composites, a material jetting 3D printer integrated with a magnetic array-based particle alignment system was developed and utilized. The material jetting device was controlled using the Labview computing environment (National Instruments, Austin, TX, USA). The sample geometry, designed using Solidworks (Dassault Systems, Veliz-Villacoublay, France), was further processed using the open-source software Slic3r to generate the G-code for the nozzle deposition path. The generated G-code was further modified to accommodate particle alignment steps after every deposited layer.

### 3.3. Characterization of Magnetic Polymer Composites

Using scanning electron microscopy (SEM), the morphology of the magnetic particle reinforced composites was analyzed with a Zeiss Sigma 300 VP field emission scanning electron microscope (Oberkochen, Germany). The composite samples were cut, polished and coated with carbon. The latter was accomplished using a Leica EM SCD005 evaporative carbon coater (Wetzlar, Germany).

Thermogravimetric analysis (TGA) was used as a tool to evaluate the resultant magnetic filler loading in the manufactured magnetic polymer composites. TGA was conducted using a Discovery TGA (TA Instruments, Delaware, USA) from 25 °C to 600 °C in a nitrogen atmosphere at a flow rate of 20 mL/min and at a heating rate of 20 °C/min.

X-ray diffraction (XRD) analysis was utilized to characterize the orientation of the easy axis of magnetization in the manufactured magnetic composites. XRD measurements were performed using a Geigerflex diffractometer (Rigaku Corporation, Tokyo, Japan) fitted with a cobalt tube as an X-ray source and a graphite monochromator to filter the K_β_ wavelength. Tests were conducted at 38 kV and 38 mA, and the samples were scanned over 2θs ranging from 30° to 70° at a rate of 2°/min.

Fourier transform infrared spectroscopy (FTIR) analysis was conducted to evaluate the degree of monomer conversion in the composites fabricated using the dual-cure acrylate-based prepolymer. FTIR analysis was conducted using a Nicolet iS50 spectrometer (Thermo Fisher Scientific, Waaltham, MA, USA). FTIR spectra were collected over a range from 400 cm^−1^ to 4000 cm^−1^ at a resolution of 4 cm^−1^. The uncured prepolymer was also characterized to compare its peak intensities with the cured polymers, which is indicative of the degree of monomer conversion.

The magnetic properties of fabricated composites were measured using a vibrating sample magnetometer (VSM, Versa Lab, Quantum Design, San Diego, CA, USA), in two conditions: parallel to the direction of field (in-plane) and perpendicular to the direction of field (out-of-plane). A small sample piece was sectioned using a handsaw, weighed and placed in the brass sample holder. For in-plane measurements, samples were secured between two quartz braces. For the out-of-plane measurement, a small amount of GE 7031 varnish and Kapton tape were also used. The effect of quartz, GE varnish and Kapton tape on the measurements is negligible. Magnetic hysteresis loops were derived by applying a magnetic field with a strength of µ_0_*H* = ± 3 Tesla at a temperature of 300 K. The magnetic properties were determined by averaging the values obtained through both magnetization and demagnetization cycles.

## 4. Results and Discussion

### 4.1. Evaluation of Dual-Cure Resin Formulation

**Figure 1:**
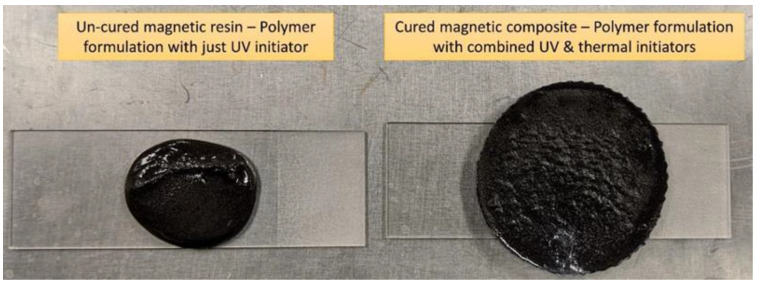
Thermal cure evaluation of dual-cure prepolymer reinforced with magnetic fillers.

**Figure 2:**
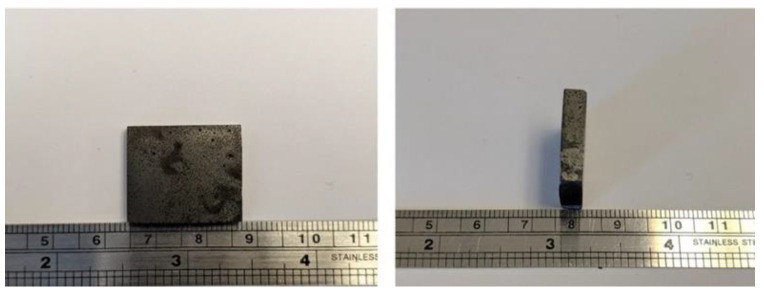
Cut and ground sample of cured magnetic composite created using dual-cure prepolymer reinforced with magnetic fillers.

UV-curable resin formulations require UV for the reaction initiation step. Free radicals are only generated during UV irradiation which enable reaction propagation and crosslinking of the monomers. One of the challenges with magnetic particle reinforced UV curable prepolymers is to achieve complete solidification as a result of polymerization. UV curable prepolymers reinforced with magnetic fillers experience lower levels of monomer conversion due to opacity introduced by the magnetic fillers. In addition to free radical generation through photochemical methods, a heat-initiated monomer conversion was introduced by adding the AIBN thermal initiator to the polymer formulation [33]. Initiator loading fractions ranging from 0.1 wt% to 0.4 wt% were tested. Adding AIBN at 0.4 wt% to a formulation containing magnetic fillers at 80 wt% was observed to render a cured magnetic polymer composite. Polymer casting was adopted for the initial trials. The prepolymer was observed to cure within an hour at temperatures ranging from 50 °C to 70 °C. A temperature range is indicated to account for any fluctuations within the oven. Initial results shown in Figure 1 indicate the ability of the material to fully cure through heat-initiated free radical polymerization. The cured composite was cut and smoothed using emery cloth for further observations (see photographs in Figure 2) exhibiting some voids in a solid material.

### 4.2. Manufacturing Field Structured Magnetic Composites Using Polymer Casting and Additive Manufacturing

**Figure 3:**
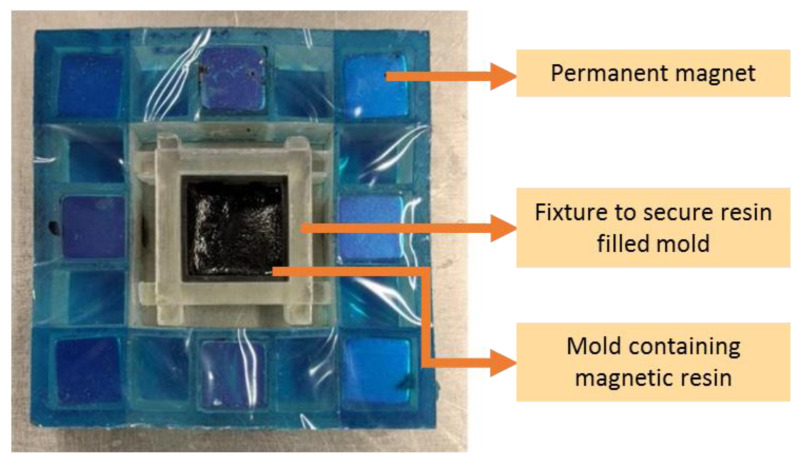
Magnetic alignment setup with permanent magnet array used to develop field structured magnetic composites.

The magnetic alignment setup utilized to develop field structured magnetic composites was composed of a magnetic array with eight cube-shaped permanent magnets arranged in an orientation to deliver a uniaxial magnetic field. In a previous study, through the finite element method in magnetics, it was observed that the magnitude of magnetic flux density produced by the magnetic array was close to 0.3 Tesla [34]. The development of the uniaxial magnetic field within the magnetic array was further confirmed using optical microscopy where particle alignment within a magnetic particle reinforced resin droplet was evaluated [34]. The photograph in Figure 3 shows the setup prepared for manufacturing field structured magnetic composites. Securing the mold filled with the magnetic particle reinforced prepolymer was critical as the mold lost its stability within the magnetic array due to the magnetic forces. The fixture to hold the alignment magnets in the magnetic array, the mold securing fixture, and the resin holding mold were all 3D printed using the Autodesk Ember Digital Light Processing (DLP) 3D printer (Autodesk Inc., San Rafael, CA, USA). Using this method, magnetic composites with the dual-cure prepolymer and epoxy-based prepolymer were fabricated and tested.

**Figure 4:**
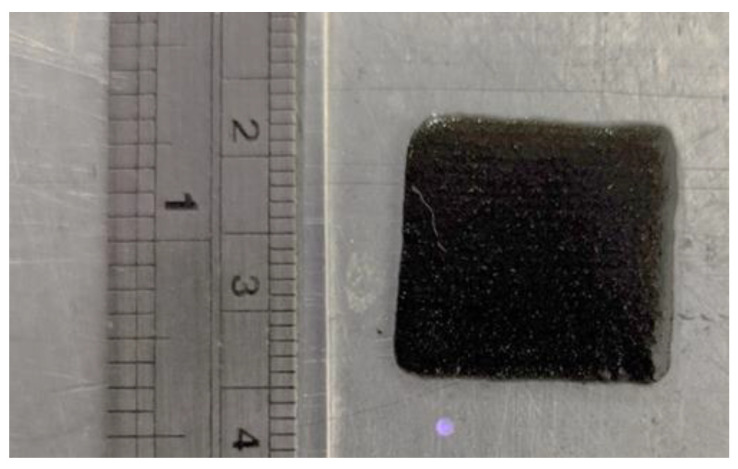
Single material layer deposited using the material-jetting 3D printer.

**Figure 5:**
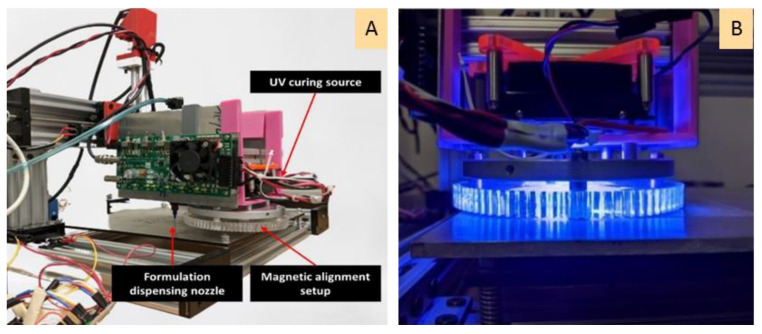
(**A**) Material jetting equipment with the in-situ particle alignment system and (**B**) UV source irradiating polymer sample for curing.

Following the polymer casting trials, the dual-cure resin formulation was utilized to 3D print magnetic composites using the material jetting AM process. The process parameters of deposition pressure and speed were adjusted to 3 kPa and 10 mm/s, respectively. Thickness settings were 0.35 mm for the initially deposited layer and 0.5 mm for subsequent layer deposition. The photograph in Figure 4 shows a single deposited layer on an acrylic sheet substrate. Note that UV curing of the deposited layer in conjunction with particle alignment using the magnetic array was accomplished using ‘manufacturing scenario B’ established in a previous publication by the present authors [35]. In summary, the process step for each layer involves (i) material deposition, (ii) magnetic particle alignment for a set time, and (iii) UV exposure for 60 s. The photographs in Figure 5 show the material jetting equipment with the in-situ particle alignment system and the UV source for subsequent curing.

**Figure 6:**
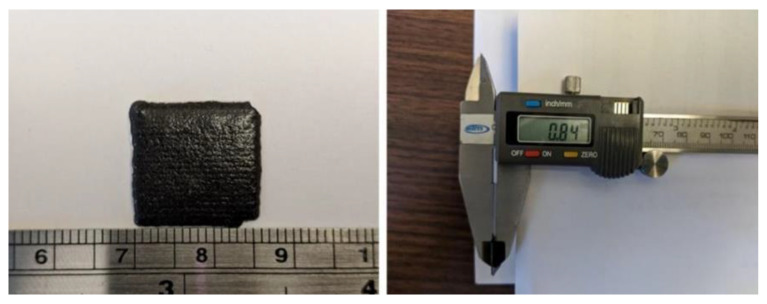
Appearance and dimensions of a single cured layer of 3D printed magnetic composite.

**Figure 7:**
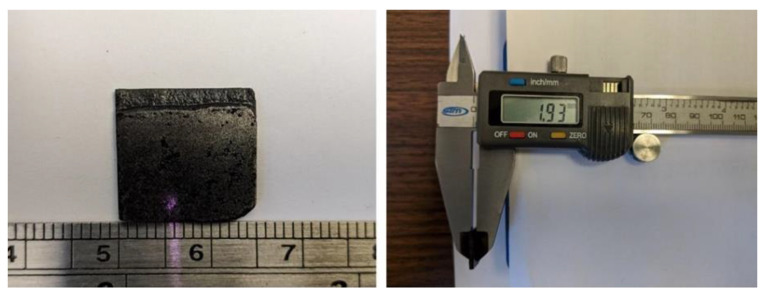
Appearance and dimensions of a cut and smoothed 3D printed magnetic composite with three print layers.

After completion of the thermal cure, the thickness of a single cured layer was measured using a digital caliper as shown in the photographs in Figure 6. Notably, with 0.84 mm the final thickness for the single-layer sample exceeded substantially the respective process parameter (0.35 mm). For multilayer samples the process of prepolymer deposition, particle alignment and UV curing was repeated, followed by thermal curing of samples. The photographs in Figure 7 show a 3D printed and cured sample consisting of three layers. The sample was cut and smoothed using emery cloth, leading to a slightly reduced sample thickness. Again, the measurement indicated in the figure reveals a final sample thickness that considerably exceeds the nominal thickness based on process parameters (i.e., 1.93 mm > 0.35 mm + 2 × 0.5 mm = 1.35 mm). Clearly, the used process parameters were not adjusted for print accuracy when using the filler modified prepolymer, which is considered a task beyond the scope of the work presented herein.

### 4.3. Characterization of Magnetic Polymer Composites

This section details the tests that were conducted to characterize the properties of fabricated magnetic composites (see Table 1), including composite morphology, filler loading, monomer conversion and magnetic anisotropy.

#### 4.3.1. Scanning Electron Microscopy

**Figure 8:**
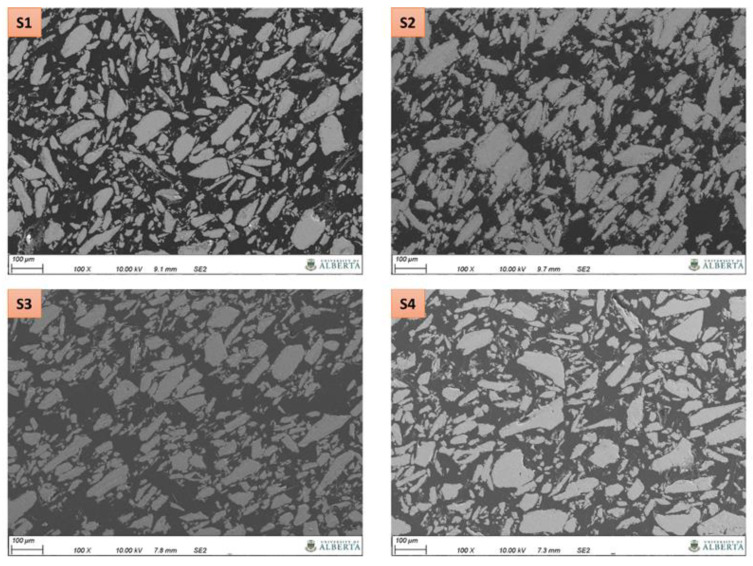
SEM images of magnetic composites as listed in Table 1.

**Figure 9:**
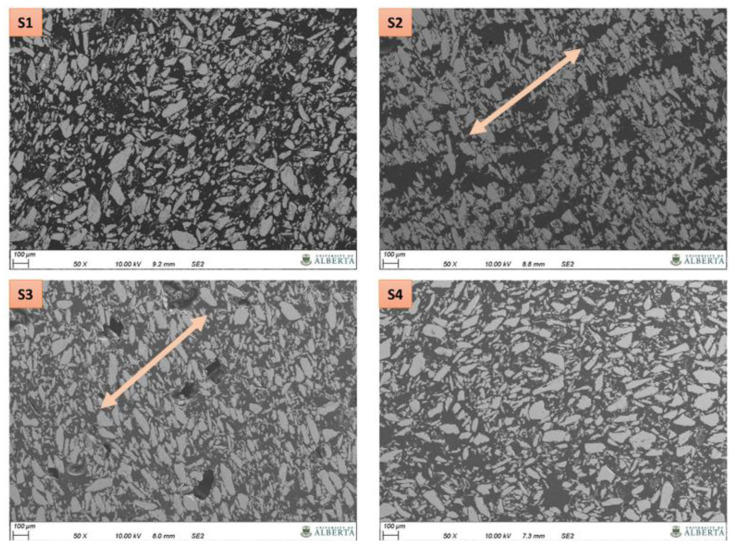
SEM micrographs of magnetic composites as listed in Table 1. Arrows indicate apparent particle stacking and alignment.

The morphology of composites consisting of magnetic particles in a polymer matrix was explored using SEM. The fundamental goal of the SEM study was to identify specific filler distribution and/or alignment characteristics due to the externally applied magnetic field. SEM micrographs of magnetic composites taken at two different magnifications are shown in Figure 8 and Figure 9. In the samples manufactured via polymer casting (S2 and S3) coupled with the magnetic field source, the SEM images exhibit particles structured in a specific direction. Arrows included in Figure 9 indicate apparent particle stacking and alignment. Such striations irrespective of the particle size elucidate the influence of an externally applied magnetic field. Similar microstructural features for field structured magnetic composites were observed by Gandha et al. [31] for anisotropic NdFeB powder reinforced polyamide, where the magnetic filler was aligned using a post printing alignment field of 1 Tesla. Differences in the apparent degree of particle alignment and stacking between sample S2 and S3 in Figure 9 are likely related to the prepolymer viscosities. From previous research it is known that the viscosity of the acrylate-based prepolymer is approximately 0.3 Pa·s whereas the value for the epoxy resin is 7 Pa·s [20,35].

Alignment or stacking features are not apparent in Figure 9 for the sample fabricated without application of an external magnetic field source (S4), suggesting an isotropic composite morphology. Referring to sample S1 that was 3D printed using material jetting and an applied magnetic field source, many particles appear to exhibit structuring similar to samples S2 and S3, yet the microstructure also exhibits features similar to the isotropic magnetic composite. Given this ambiguity, it is difficult to confirm whether or not alignment or stacking features are due to the externally applied magnetic field or other effects (e.g., occupied volume effects). It should be mentioned that for the S1 sample the magnetic particle alignment time was shorter than for the samples produced by polymer casting. Adjusting the alignment time for 3D printed samples was necessary to mitigate material deformation under the influence of the magnetic field because no mold is providing material containment in this case (as discussed in [35]).

Besides morphological features relating to particles participles, resin rich spaces and porosities can be observed in the manufactured samples in Figure 8 and Figure 9. These observations suggest that increased particle loading is feasible and that process adjustments need to be made to reduce porosity, with the latter being outside the scope of this paper.

#### 4.3.2. Thermogravimetric Analysis

**Figure 10:**
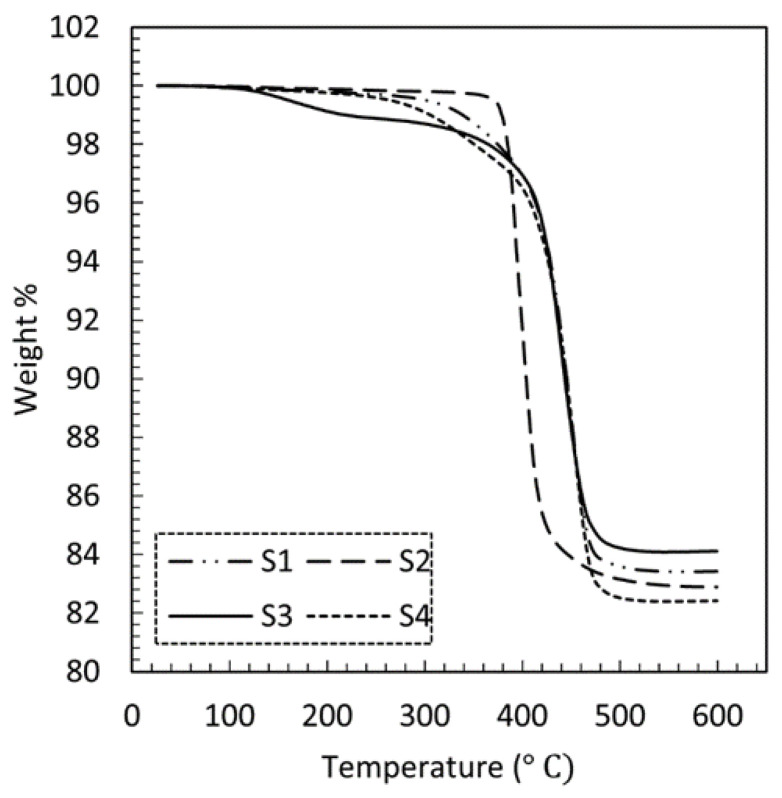
TGA data of magnetic composites as listed in Table 1.

TGA was used to determine the magnetic particle loading in the fabricated magnetic composites. The thermal resin removal enabled determining the remaining weight of the sample in the crucible. The experimental data obtained from the TGA tests are depicted in Figure 10. The step transition temperature for samples S1, S2, S3, and S4 were observed to be 411 °C, 380 °C, 407 °C, and 413 °C, respectively. The percentage weight at 600 °C (end of the test) was taken as the weight percentage of the remaining magnetic filler material in the polymer composite. Table 2 lists the percentage weight of the residue at different temperatures obtained from TGA testing. It can be observed that the residue weight percentage of the magnetic filler exceeds the nominal filler weight fraction of the prepolymer formulation (80 wt%) by a maximum of about 5%. It is speculated that this discrepancy is rooted in material handling and manufacturing processes where effects such as material settling may have led to a slight increase in final filler weight fraction.

#### 4.3.3. X-ray Diffraction Analysis

**Figure 11:**
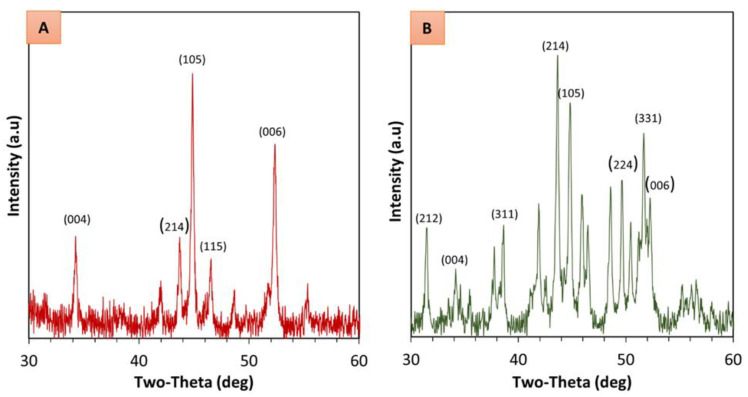
XRD patterns of (**A**) anisotropic magnetic composite fabricated with external magnetic field application, and (**B**) isotropic magnetic composite fabricated without an external magnetic field.

X-ray diffraction was used to identify the orientation of the easy axis of magnetization in the composites as a result of the externally applied magnetic field. XRD analysis was conducted only for sample S2 and an additional isotropic sample fabricated using epoxy as the base prepolymer. In the presence of a magnetic field, a magnetic moment is generated along the easy axis of magnetization which results in particles interacting and forming aligned microstructures [35]. First, the magnetic powder was characterized to identify and confirm the chemical composition. The obtained XRD data was matched to the Powder Diffraction File reference data for NdFeB (number 00-039-0473). The 2θ incident angles representing the crystallographic c-axis were identified to facilitate comparisons for isotropic and anisotropic magnetic composites. Corresponding XRD data for 2θ incident angles ranging between 30° to 60° are depicted in Figure 11. From technical literature, it is understood that for NdFeB an enhancement of intensity of the (006) and (004) crystallographic plane indicates the orientation of the easy axis of magnetization in the magnetic composites [36]. Additionally, the disappearance of dominant peaks typically observed for a magnetic composite with an isotropic particle distribution is seen in Figure 11A. Results observed herein are congruent with the findings reported in the technical literature for field structured magnetic composites [31], and hence, an aligned microstructure can be ascertained for the S2 sample that was exposed to the magnetic field during manufacturing.

#### 4.3.4. FTIR Spectroscopy

**Figure 12:**
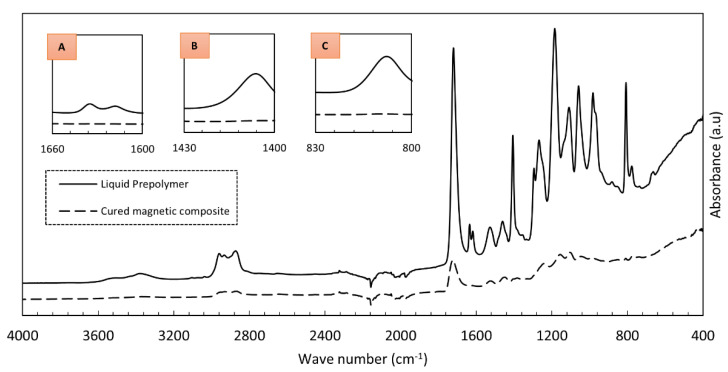
FTIR spectra of uncured liquid prepolymer and cured magnetic composite. Insets indicate FTIR spectral regions: (**A**) 1600 cm^−1^ to 1660 cm^−1^, (**B**) 1400 cm^−1^ to 1430 cm^−1^, and (**C**) 800 cm^−1^ to 830 cm^−1^.

FTIR spectra of the developed dual-cure liquid prepolymer and the corresponding magnetic composite cured by UV and heat are shown in the graph in Figure 12. Note that the composite sample was in the form of a crushed powder. The obtained spectra were inspected primarily for reductions in peak intensities (i.e., peak flattening) that directly correlate to monomer conversion in the polymer composite [37,38]. Signals that correspond to the carbon double bond (C=C) in the acrylate along the regions 800 cm^−1^ to 830 cm^−1^ (=C-H out of plane bend), 1400 cm^−1^ to 1430 cm^−1^ (C=C twisting), and 1600 cm^−1^ to 1660 cm^−1^ (C=C stretching) were observed to diminish in the cured polymer composite compared to the uncured prepolymer, indicating that the composite was cured under the influence of heat in addition to UV irradiation [39].

#### 4.3.5. Magnetic Characterization

**Figure 13:**
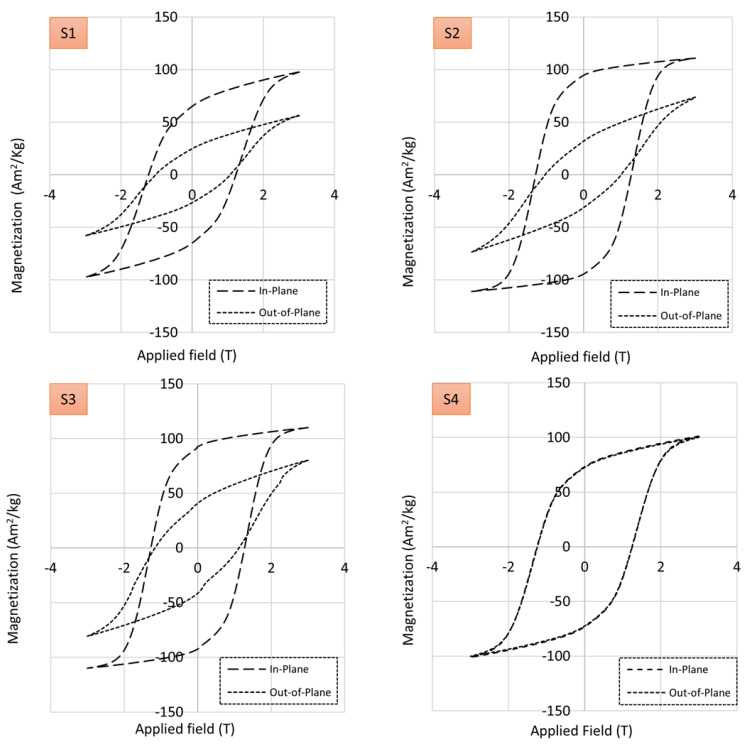
Hysteresis loops of magnetic composites as listed in Table 1, measured in sample in-plane and out-of-plane orientation using a vibrating sample magnetometer.

Composites reinforced with magnetic particles can be characterized by their magnetic saturation, remanence, and coercivity. Composites containing hard ferromagnetic particles should generate sufficient magnetic flux for a given application once they are magnetized, i.e., energized by applying a magnetic field using electromagnets. However, the properties of magnetic composites are dependent on microstructure, temperature, and demagnetizing fields [31]. The graphs in Figure 13 depict hysteresis loops measured using the vibrating sample magnetometer along the direction of particle structuring (in-plane) and perpendicular to the direction of particle structuring (out-of-plane). The samples fabricated applying an external magnetic field (S1, S2 and S3) exhibit significant enhancements in magnetic properties (saturation magnetization, squareness, and coercivity) along the in-plane easy direction over the out-of-plane direction. The presence of an applied magnetic field can be clearly seen to result in a preferred magnetization direction and strong anisotropy. This magnetic anisotropy is conferred by the preferential alignment of the magnetic filler powders along the curing applied field, as evidenced in Figure 8, Figure 9 and Figure 11. Field-structured magnetic composites developed using polymer casting (S2 and S3) exhibited anisotropic magnetic properties comparable to, or greater than, the isotropic specimen (S4). For the latter, no distinguishable differences can be ascertained between the in-plane and out-of-plane hysteresis loops. While the 3D printed sample also exhibits enhanced in-plane properties over the out-of-plane direction, it needs to be noticed that its properties are lower compared to the other samples. For example, the remanence to saturation ratio, which characterizes the degree of anisotropy, was observed to be approximately 0.85 for samples fabricated using polymer casting and 0.67 for the 3D printed sample. As mentioned earlier, a lower particle alignment time was employed for 3D printed samples (to mitigate magnetic resin deformation [35]), which is seen to have caused the somewhat reduced magnetic properties. Nevertheless, even with the reduced alignment time, the composite exhibited pronounced anisotropic and in-plane magnetic properties comparable to the other samples.

**Figure 14:**
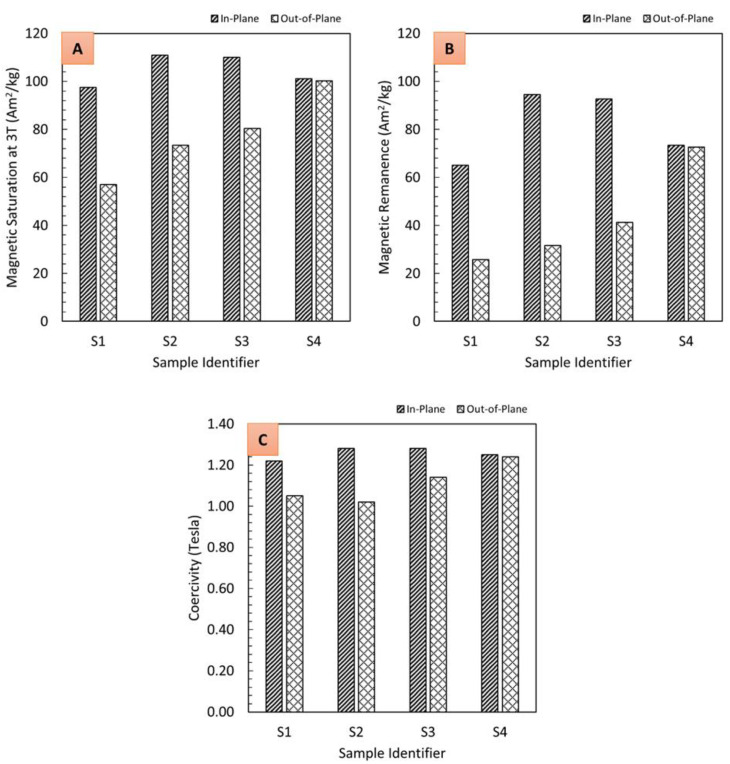
In-plane and out-of-plane magnetic properties of composites as listed in Table 1, derived considering magnetization and demagnetization cycles of hysteresis data: (**A**) magnetic saturation at 3 Tesla, (**B**) remanence, and (**C**) coercivity.

Data derived from magnetization and demagnetization cycles in the hysteresis graphs in Figure 13 are reduced to key properties in terms of magnetic saturation (at 3 Tesla), remanence, and coercivity in the bar graphs in Figure 14. The bar graphs indicate the efficacy of field structuring to achieve higher magnetic properties, i.e., saturation, remanence, and coercivity for the in-plane direction are consistently higher for samples S2 and S3 compared to the isotropic sample S4. Present observations are congruent to magnetic composites containing field structured particles in a thermoplastic matrix [31,32]. Overall, the present study validates the effectiveness of field structuring magnetic particles in thermoset polymers using a permanent magnet array, enabling an efficient fabrication of anisotropic magnetic composites for a variety of applications in electrical and electronic devices.

## 5. Conclusions

In this study, magnetic composites with anisotropic and isotropic properties were created using two different types of prepolymer formulations, i.e., a dual-cure (via UV irradiation and heat) acrylate-based resin and a commercially available heat curable epoxy. Both prepolymers were employed in a polymer casting process, while the dual-cure resin was also utilized in a material jetting additive manufacturing approach. It was demonstrated that the thermal initiator added to the UV curable prepolymer rendered the formulation to also be heat curable. A thermal initiator loading of 0.4 wt% was observed to solidify the prepolymer reinforced with approximately 80 wt% NdFeB magnetic particles. Molds made using stereolithography 3D printer enabled holding the resin during the magnetic alignment and thermal curing processes. The developed dual-cure prepolymer formulation was shown to be 3D printable using in-house developed material jetting AM equipment. It was observed that the thickness of cured composite layers was greater than what would be expected for the given process parameters and an unreinforced prepolymer. SEM images of magnetic composites revealed a material morphology with filler alignment and stacking features indicating directional orientation of magnetic particles in specimens fabricated using the polymer casting approach. Irrespective of the type of prepolymer used, the microstructures were highly similar. Conversely, casting the dual-cure prepolymer without the magnetic alignment process resulted in a composite sample with isotropic appearance. The microstructure of the sample fabricated using material jetting and magnetic alignment appeared to feature some particle alignment and stacking while also having similarities to the isotropic morphology. The observed morphology was attributed to a lower magnetic alignment time utilized in the additive manufacturing approach.

The ability of the permanent magnet array to orient magnetic particles in the prepolymer was confirmed through X-ray diffraction analysis where enhancements in peak intensities corresponding to the alignment of the easy axis of magnetization were observed. Thermogravimetric analysis enabled determining the magnetic filler loading in the polymer composites by conducting a thermal resin removal. From FTIR spectroscopy, through observations of peak flattening along the carbon double bond regions, it was confirmed that the UV curable resin modified with the thermal initiator was polymerized under the influence of heat.

Composites characterized for magnetic properties using a vibrating sample magnetometer revealed an enhancement in magnetic properties along the in-plane direction, i.e., the direction of magnetic field structuring, compared to the out-of-plane directions. Magnetic saturation within the tested applied magnetic field range, remanence, and coercivity were all observed to be enhanced along the in-plane direction. Samples with magnetic filler alignment and fabricated through the polymer casting approach were observed to exhibit the highest magnetic characteristics. Even though microscopy of the 3D printed sample did not reveal strong filler alignment, anisotropic magnetic properties were ascertained, albeit lower than for the samples produced by polymer casting.

This research validated the efficacy of magnetic field induced alignment of a magnetic filler for the fabrication of magnetic thermoset composites. The use of a dual-cure thermoset resin further enabled material jetting additive manufacturing using UV irradiation for pre-curing the prepolymer during printing, followed by post-processing via heat curing to ensure full polymerization of the opaque material formulation. Future work shall encompass process optimization to further enhance magnetic properties, improve dimensional accuracy, and mitigate unwanted morphological features such as resin rich zones and voids. Ultimately, this research has provided a pathway to combine a dual-cure resin formulation along with magnetic alignment and additive manufacturing to construct anisotropic magnetic composites for applications in electrical and electronic devices.

## Figures and Tables

**Table 1:** List of fabricated and characterized sample types.

Sample	Material	Manufacturing Process	Magnetic Alignment
S1	MQA-38-14 dispersed in dual-cure prepolymer	Material-jetting AM	Yes
S2	MQA-38-14 dispersed in heat-curable epoxy resin	Polymer casting	Yes
S3	MQA-38-14 dispersed in dual-cure prepolymer	Polymer casting	Yes
S4	MQA-38-14 dispersed in dual-cure prepolymer	Polymer casting	No

**Table 2:** Residue weight percentage at different temperatures for composites as listed in Table 1.

Sample	Residue wt% at 200 °C	Residue wt% at 600 °C
S1	99.7	83.4
S2	99.8	82.9
S3	99.1	84.1
S4	99.7	82.4

## Data Availability

The data that support the findings of this study are available from the corresponding author upon reasonable request.
